# Exploring the effect of canine cancer-associated fibroblasts on T cell dynamics through the CXCL12/CXCR4 axis modulated by TGF-β1

**DOI:** 10.1038/s41598-025-16312-x

**Published:** 2025-08-23

**Authors:** Ayano Kudo, Shintaro Kamo, Akinori Yamauchi, Sho Yoshimoto, Yuma Harada, Eiichi Kanai, Satoshi Takagi

**Affiliations:** 1 Laboratory of Small Animal Surgery, School of Veterinary Medicine, Azabu University, 1-17-71 Fuchinobe, Chuo-ku, Sagamihara, Kanagawa 252-5201 Japan; 2 Azabu University Veterinary Teaching Hospital, Azabu University, 1-17-71 Fuchinobe, Chuo-ku, Sagamihara, Kanagawa 252-5201, Japan

**Keywords:** Cancer-associated fibroblasts, Tumor microenvironment, Dog, C-X-C motif chemokine ligand 12, C-X-C chemokine receptor 4, Transforming growth factor-β1, Cancer microenvironment, Tumour immunology, Chemotaxis

## Abstract

Cancer-associated fibroblasts (CAFs) are key components of the tumor microenvironment (TME) that modulate T cell immunity by secreting humoral factors and forming structural barriers. CAFs secrete the chemokine C-X-C motif chemokine ligand 12 (CXCL12), which binds to C-X-C chemokine receptor 4 (CXCR4) on T cells and induces chemotaxis. Transforming growth factor beta 1 (TGF-β1), another humoral factor secreted by CAFs, has been reported to regulate the CXCL12/CXCR4 axis; however, a direct association between them has not been demonstrated in human medicine or veterinary medicine. This study investigated the role of canine CAFs in T cell migration through the CXCL12/CXCR4 axis and the regulatory influence of TGF-β1. CXCL12 and CXCR4 were expressed in the tumor stroma and on T cells, respectively, in dogs with epithelial malignant tumors. Canine CAFs secreted higher levels of CXCL12 and TGF-β1 than normal fibroblasts, and CAF-derived TGF-β1 modulated both CXCL12 secretion by CAFs and CXCR4 expression on T cells. Furthermore, canine CAFs induced T cell migration through the CXCL12/CXCR4 axis. These findings indicate that CAFs may influence T cell migration through the CXCL12/CXCR4 axis under the regulation of TGF-β1 signaling, highlighting their potential role in shaping T cell dynamics within the TME.

## Introduction

The tumor microenvironment (TME) is a highly intricate structure comprised of tumor and stromal cells, including immune cells, endothelial cells, and fibroblasts. Among these, cancer-associated fibroblasts (CAFs) are recognized as a principal component of the tumor stroma^1^. CAFs enhance tumor malignancy by promoting angiogenesis, proliferation, invasion, and metastasis. Notably, their role in modulating T cell immunity has garnered significant attention. The inhibition of T cell infiltration into solid tumors by CAFs results from the secretion of various humoral factors and their function as a physical barrier, characterized by dense extracellular matrix fiber networks^2,3^.

The C-X-C motif chemokine ligand 12 (CXCL12) is one of the chemokines secreted by CAFs^2^,and it interacts with the C-X-C chemokine receptor 4 (CXCR4) on T cell membranes, inducing chemotaxis, cell proliferation and gene expression^3,4^. High expression levels of CXCL12 in the tumor stroma have been detected in various human cancers, including gastric, bladder, and ovarian cancer through immunohistochemistry (IHC), and CXCL12 upregulation correlated with poor prognosis^5–7^. In CXCL12-positive stroma, T cells are more abundant in the stromal area than in the tumor nests. This shows that CXCL12 retains T cells in the stroma and limits their access to cancer cells, thereby impairing antitumor immunity and potentially contributing to poor prognosis^7,8^. In vitro studies have further demonstrated that human CAFs secrete higher levels of CXCL12 than normal fibroblasts (NFs)^5,9,10^ and that these CAFs promote T cell migration through CXCL12^9^. As for the receptor CXCR4, CD8^+^ T cells exhibited higher CXCR4 expression in patients of pancreatic ductal adenocarcinoma compared with healthy individuals^9^. Considering these findings, the combination of CXCL12/CXCR4 inhibitors with an immune checkpoint inhibitor has been examined in mouse models and shown to significantly enhance antitumor effects^8,11,12^. This strategy is currently being explored in human medicine, with phase I-II clinical trials underway.

Transforming growth factor beta (TGF-β), another humoral factor secreted by CAFs, has been implicated to modulate the CXCL12/CXCR4 axis. Human CAFs secrete higher levels of TGF-β than NFs^13^, and TGF-β mediated autocrine or paracrine signaling activates CAFs, increasing CXCL12 secretion^13,14^. Previous studies have reported that TGF-β isoforms increase CXCR4 expression on human CD4^+^ T cells^15^ and similarly the culture supernatant of human CAFs upregulates CXCR4 expression on T cells^16^. Although the relationship between CXCR4 expression and CAF-derived TGF-β remains unclear, these findings indicate that CAFs modulate T cell migration within the TME by regulating the CXCL12/CXCR4 axis through TGF-β. However, direct evidence for this interaction has not been reported.

In veterinary medicine, research on the biology of CAFs remains limited. Several studies isolating canine CAFs from epithelial malignancies have shown that canine CAFs share functional similarities with human CAFs, including high expression of alpha smooth muscle actin (α-SMA), a marker of activated fibroblasts, and promotion of tumor cell migration and invasion through humoral factors^17–19^. Elevated serum CXCL12 levels have been observed in dogs with metastatic mammary carcinoma relative to healthy dogs^20^. In addition, serum TGF-β levels are have also been reported to be significantly higher in tumor-bearing dogs than in healthy controls^21^. However, the relationship between these elevated serum factors and the presence of CAFs remains unclear in veterinary medicine. Although stromal markers and soluble factor-related DNA have been tried to investigate in canine mammary gland carcinoma tissues^22^, the CXCL12/CXCR4 axis in canine CAFs has not been studies, and its role in regulating T cell migration remains largely unexplored.

This study aimed to examine the influence of canine CAFs on T cell migration through the CXCL12/CXCR4 axis and elucidate the mechanism underlying TGF-β-mediated regulation of this axis by CAFs, an area that remains underexplored in both veterinary and human medicine.

## Results

### CXCL12 expression in the stroma of canine epithelial malignant tumors

**Fig. 1:**
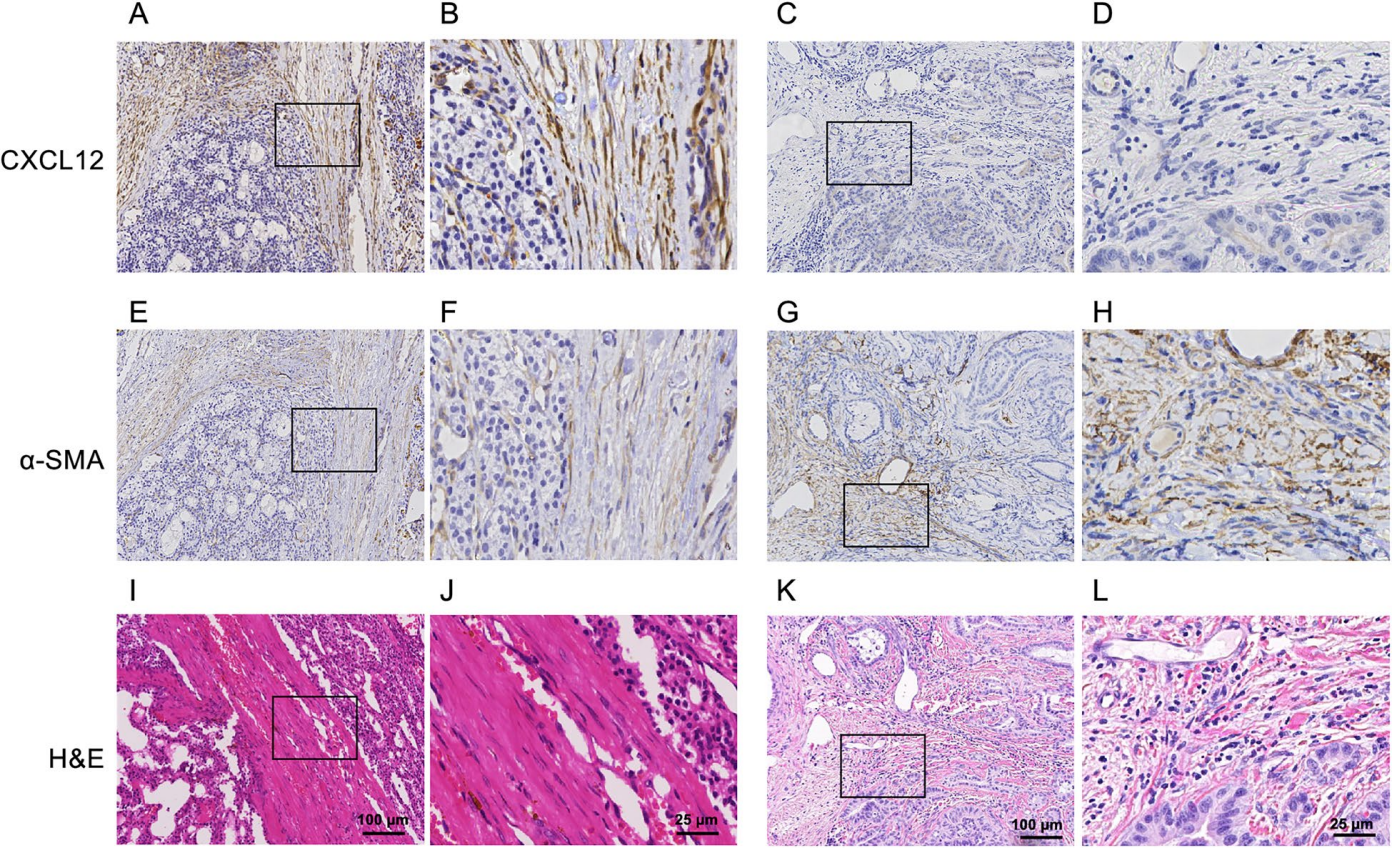
IHC of CXCL12 and α-SMA. (**A**–**D)** CXCL12, (**E**–**H**) α-SMA, (**I**–**L**) Hematoxylin and eosin staining. *Left two columns* canine thyroid carcinoma tissue with a CXCL12 score of 3; *Right two columns* canine lung adenocarcinoma tissue with a CXCL12 score of 0. The left view of each case is at a low power field, and the right view is at a high-power field.

CXCL12 expression in the tumor stroma was investigated in 70 tissue samples of canine epithelial malignant tumors. CXCL12 expression was detected in the cytoplasm of stromal cells adjacent to tumor cells (Fig. 1A–D). Among the tumor types, CXCL12 expression was detected in cases of thyroid carcinoma (7/10), renal cell carcinoma (7/10), intestinal adenocarcinoma (3/5), prostate adenocarcinoma (5/10), hepatocellular carcinoma (2/5), mammary gland carcinoma (2/5), lung adenocarcinoma (3/10) and transitional cell carcinoma (2/10) (Table 1). The CXCL12 expression score for thyroid carcinoma and renal cell carcinoma tended to be higher than that for other tumors. Fibroblasts in the tumor stroma were positive for α-SMA in all cases (Fig. 1E–H). Hematoxylin and eosin staining was performed to confirm the tumor and stromal regions (Fig. 1I–L). Supplementary Fig. S1 shows stromal CXCL12 expression in the remaining tumor histo-types, including both positive and negative cases.

**Table 1:** CXCL12 expression in various epithelial malignant tumors.

Pathology	Positive no./tested no	Positive rate (%)	CXCL12 score in tumor stroma		
			1	2	3
Thyroid carcinoma	7/10	70	2	1	4
Renal cell carcinoma	7/10	70	2	2	3
Intestinal adenocarcinoma	3/5	60	3	0	0
Prostate adenocarcinoma	5/10	50	3	2	0
Hepatocellular carcinoma	2/5	40	1	0	1
Mammary gland carcinoma	2/5	40	2	0	0
Lung adenocarcinoma	3/10	30	1	1	1
Transitional cell carcinoma	2/10	20	1	1	0
Anal sac gland carcinoma	0/5	0	0	0	0

CXCL12 score in tumor stroma was scored based on the following criteria: 0, no or ≤ 10% of stromal cells stained; 1, > 10% of stromal cells stained with weak intensity; 2, > 10% of stromal cells stained with moderate intensity; and 3, > 10% of stromal cells stained with strong intensity.

### Isolation of canine CAFs and detection of CXCL12 protein

**Fig. 2:**
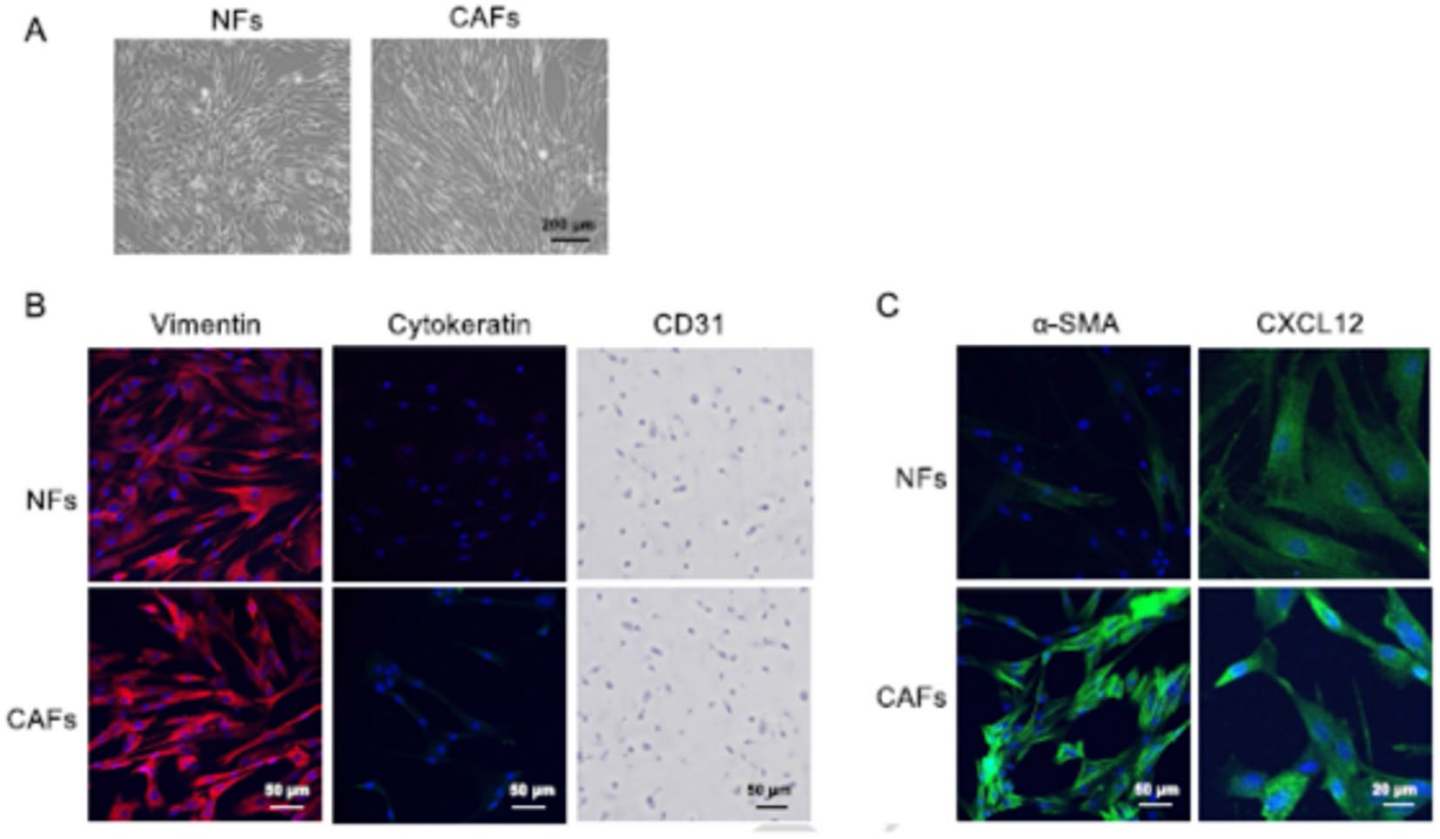
Isolation and identification of fibroblasts. (**A**) Microscopic image of NFs and CAFs isolated from a dog with lung adenocarcinoma (optical microscope). *Scale bars* 50 µm. (**B**) IF staining for vimentin (red) and cytokeratin (green), and DAB staining for CD31 (brown) in NFs (top) and CAFs (bottom) isolated from a dog with lung adenocarcinoma. Nuclei were counterstained with DAPI (blue). Vimentin and cytokeratin were visualized using a confocal microscope, and CD31 staining was observed using an optical microscope. CD31 negativity confirmed the absence of endothelial cell contamination. *Scale bars* 50 µm. (**C**) IF staining for α-SMA and CXCL12 (green) in NFs (top) and CAFs (bottom). Nuclei were counterstained with DAPI (blue). *Scale bars* 50 µm (α-SMA), 20 µm (CXCL12).

Primary cultures of canine CAFs were established from surgically resected tumor tissues obtained from 10 dogs diagnosed with epithelial malignant tumors. The patient characteristics are summarized in Supplementary Table S1. NFs were also isolated from the skin tissue of the same patients; however, primary cultures could not be successfully established in all cases. The cells obtained were purified by passaging once or twice after the initial culture, and each cell had elongated, spindle-shaped fibroblast features (Fig. 2A). Fibroblasts were identified based on positive staining for vimentin and negative staining for cytokeratin and CD31 (Fig. 2B). CAFs showed positive staining for α-SMA, while NFs showed weak expression in only a subset of cells (Fig. 2C). CXCL12 expression in CAFs was higher than that in NFs, with particularly strong expression observed around the nucleus.

### TGF-β1-mediated regulation of CXCL12 secretion from canine CAFs

**Fig. 3:**
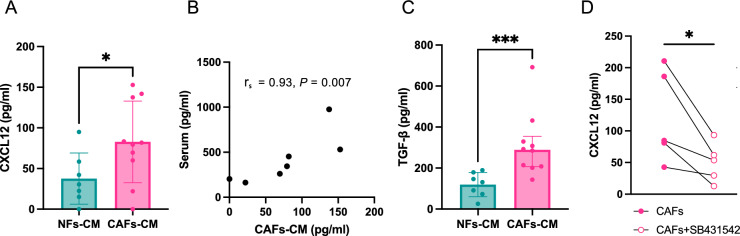
Evaluation of CXCL12 and TGF-β1 secretion from CAFs. (**A**) CXCL12 concentrations in NF-CM and CAF-CM. Data are presented as mean ± SD (NF-CM, *n* = 7; CAF-CM, *n* = 10). (**B**) Scatter plot showing the correlation between CXCL12 levels in CAF-CM and serum CXCL12 concentrations, with Spearman’s rank correlation coefficient (rₛ) and *P* value indicated (*n* = 7). (**C**) TGF-β1 concentrations in NF-CM and CAF-CM. Data are presented as median with IQR (NF-CM, *n* = 7; CAF-CM, *n* = 10). (**D**) CXCL12 secretion from CAFs cultured in the presence of the TGF-βRI inhibitor SB431542 (*n* = 5). *P < 0.05, ***P < 0.001.

CXCL12 protein levels in culture media from canine NFs (NF-CM) and CAFs (CAF-CM) were quantified using enzyme-linked immunosorbent assay (ELISA). Canine CAFs secreted significantly higher levels of CXCL12 than NFs (*P* = 0.0379) (Fig. 3A). Serum CXCL12 concentrations were also evaluated in healthy dogs and in 24 dogs with epithelial malignant tumors (Supplementary Fig. S2). A significant correlation was observed between CXCL12 levels in CAF-CM and serum CXCL12 levels (rₛ = 0.9286, *P* = 0.0067) (Fig. 3B), based on matched CAFs and serum samples from the same dogs.

Next, TGF-β1 protein levels in NF-CM and CAF-CM were quantified. Canine CAFs secreted significantly higher levels of TGF-β1 than NFs (*P* = 0.0007) (Fig. 3C). A significant correlation was observed between CXCL12 and TGF-β1 levels in CAF-CM (r_s_ = 0.7091, *P* = 0.0268) (Supplementary Fig. S3).

To assess the effect of CAF-derived TGF-β1 on CXCL12 secretion, CAFs were cultured in the presence of the TGF-βRI inhibitor, SB431542. CXCL12 secretion from CAFs was significantly reduced (*P* = 0.0335) (Fig. 3D).

### Increased CXCR4 expression on peripheral circulating canine T cells in epithelial malignant tumors

**Fig. 4:**
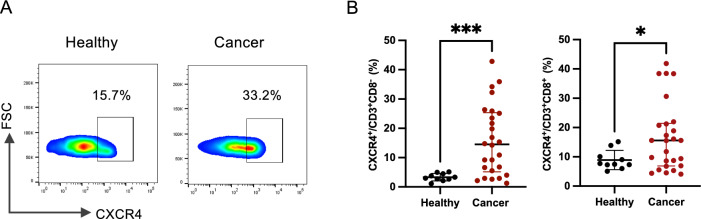
Detection of CXCR4 expression on canine T cells. (**A**) CXCR4 expression on canine CD3⁺CD8⁻ and CD3⁺CD8^+^ T cells were evaluated using flow cytometry analysis. Representative data from one healthy beagle and a cancer-bearing dog. Plots are gated on *lymphocyte* > *single cells* > *Live cells* > *CD3*^+^ *cells* > *CD8*^*-*^* or CD8*^+^ *cells*. (**B**) CXCR4 expression on CD8^+^ T cells or CD8^-^ T cells in healthy dogs and dogs with cancer. Data are presented as median with IQR (Healthy, *n* = 10; Cancer, *n* = 26). *P < 0.05, ***P < 0.001.

Surface expression of CXCR4 on peripheral circulating canine T cells was evaluated in 10 healthy dogs and 26 dogs with epithelial malignant tumors by flow cytometry. The types of epithelial malignant tumor included in this study were as follows: thyroid carcinoma (*n* = 5); renal cell carcinoma (*n* = 4); hepatocellular carcinoma (*n* = 4); lung adenocarcinoma (*n* = 3); squamous cell carcinoma (*n* = 3); anal sac gland carcinoma (*n* = 2); salivary gland carcinoma (*n* = 2); and one case each of perianal adenocarcinoma and transitional cell carcinoma. Representative data from a healthy beagle and a dog with thyroid carcinoma are shown in Fig. 4A. CXCR4 expression on CD3^+^CD8^-^ and CD3^+^CD8^+^ T cells in dogs with epithelial malignant tumors was significantly higher than that in healthy dogs (*P* = 0.0005 and *P* = 0.0402, respectively) (Fig. 4B).

### Upregulation of CXCR4 on canine T cells by CAF-derived TGF-β1

**Fig. 5:**
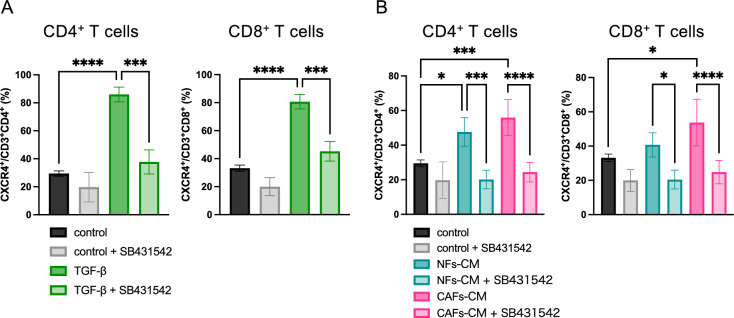
Effect of TGF-β1 on CXCR4 expression on T cells. (**A**) CXCR4 expression on CD3⁺CD4^+^ and CD3⁺CD8^+^T cells after being cultured with the medium containing TGF-β1 (5 ng/mL) with or without the TGF-βRI inhibitor SB431542 (5 µM) for 5 days. Mean ± SD (*n* = 3). Plots are gated on *lymphocyte* > *single cells* > *Live cells* > *CD3*^+^ *cells* > *CD4*^+^ *or CD8*^+^ *cells*. (**B**) CXCR4 expression on CD3⁺CD4^+^ and CD3⁺CD8^+^ T cells after being cultured with NF-CM or CAF-CM for 5 days. Mean ± SD (Control, *n* = 3; NF-CM, *n* = 5; CAF-CM, *n* = 8). *P < 0.05, ***P < 0.001, ****P < 0.0001.

The effect of TGF-β1 on CXCR4 expression in canine T cells was evaluated by culturing canine peripheral blood mononuclear cells (cPBMCs) with recombinant TGF-β1 or CAF-CM. CXCR4 expression on CD3^+^CD4^+^ and CD3^+^CD8^+^ T cells was significantly elevated by TGF-β1 stimulation (*P* < 0.0001 for both), and this upregulation was abolished by the TGF-βRI inhibitor, SB431542 (*P* = 0.0002 for both) (Fig. 5A).

Similarly, culturing with CAF-CM significantly elevated CXCR4 expression on CD3^+^CD4^+^ and CD3^+^CD8^+^ T cells (*P* = 0.0005 and *P* = 0.0303, respectively), and this effect was suppressed by SB431542 (*P* < 0.0001 for both) (Fig. 5B). In comparison, culturing with NF-CM resulted in a lower increase in CXCR4 expression than that observed with CAF-CM. However, CXCR4 expression on CD3^+^CD4 + T cells still significantly increased (*P* = 0.0405), and this effect was suppressed by SB431542.

### ***CAF-derived CXCL12 enhanced migration of canine CD8***^+^***T cells***

**Fig. 6:**
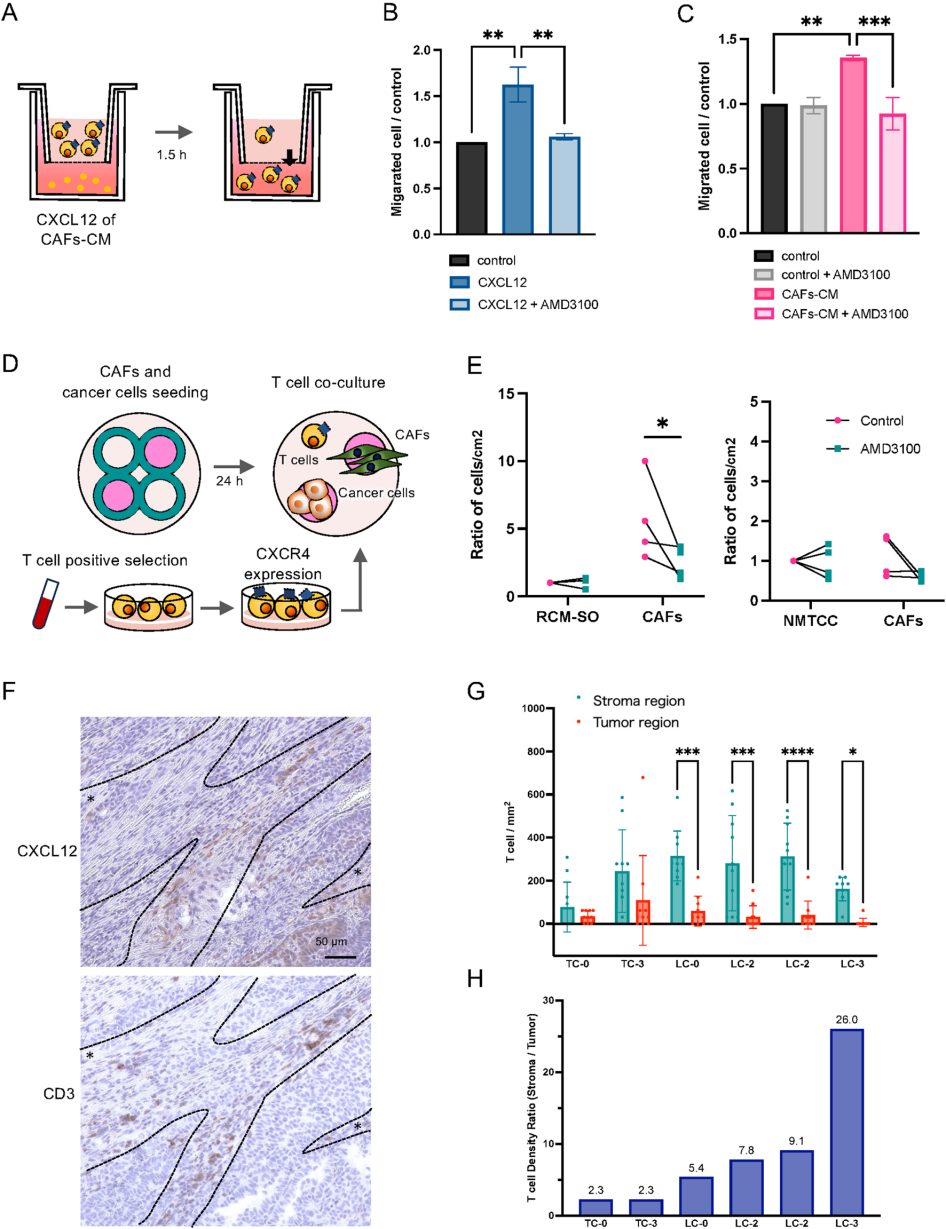
CAFs attract CD8⁺ T cells through the CXCL12/CXCR4 axis. (**A**) Schematic of the Boyden-chamber migration assay. CXCR4⁺CD8⁺ T cells were seeded in the upper compartment; the lower compartment contained control medium, recombinant CXCL12 (100 ng/mL) or CAF-CM. Migrated cells were counted after 24 h. (**B**) Migration toward recombinant CXCL12, expressed as fold change versus control. Mean ± SD (*n* = 3). (**C**) Migration toward CAF-CM, expressed as fold change versus control. Mean ± SD (*n* = 3). Control, culture medium; AMD3100, 4 µM CXCR4 inhibitor. (**D**) Schematic of the adhesion assay using a cell culture insert in a 6 cm dish. CAFs and tumor cells (RCM-SO or NMTCC) were plated on opposite sides of the insert and cultured for 24 h; the insert was then removed and CXCR4⁺ T cells added for a further 24 h. (**E**) Density of T cells adhered per cm^2^ to cancer cell lines versus CAFs after 24 h. Mean ± SD (*n* = 4). RCM-SO, canine mammary gland carcinoma cell line; NMTCC, canine transitional cell carcinoma cell line. (**F**) IHC of serial sections from a canine pulmonary adenocarcinoma stained for CXCL12 (top) and CD3 (bottom). Dashed lines delineate stromal and tumor cell areas, and asterisks (*) indicate the stromal regions. *Scale bars* 50 µm (**G**) CD3⁺ T cell density in stromal and tumor regions of thyroid carcinoma (TC,* n* = 2) and pulmonary adenocarcinoma (LC, *n* = 4). Numbers after histo-type indicate CXCL12 expression score. Mean ± SD. (**H**) Ratio of stromal to tumor CD3⁺ T cell density in each case. *P < 0.05, **P < 0.01, ***P < 0.001, ****P < 0.0001.

We determined the effect of the CXCL12/CXCR4 axis on the migration of canine CD8^+^ T cells using migration assays (Fig. 6A). CD8^+^ T cell migration towards recombinant CXCL12 was significantly higher compared with the control (Fig. 6B) (*P* = 0.0011). Pretreatment with the CXCR4 antagonist AMD3100 significantly reduced this migration (*P* = 0.0018). Moreover, the number of CD8^+^ T cells that migrated into CAF-CM was significantly higher compared with the control (*P* = 0.0013), and treatment with AMD3100 significantly abolished this increased migration (*P* = 0.0004) (Fig. 6C).

### T cells attached to CAFs rather than cancer cells through the CXCL12/CXCR4 axis

T cells, CAFs, and canine cancer cells were co-cultured (Fig. 6D). The density of attached T cells per cm^2^ on CAFs was significantly reduced when co-cultured with RCM-SO in the presence of AMD3100 (Fig. 6E) (*P* = 0.0475), whereas no significant decrease was found when co-cultured with NMTCC (*P* = 0.0955). The microscopic observation of T cells attached to CAFs is presented in Supplementary Fig. S4.

### Comparison of T cell density between stromal and tumor regions

Evaluation of serial sections stained for CD3 and CXCL12 revealed co-localization of CXCL12 expression and clusters of CD3⁺ T cells in the stromal region. Representative images of pulmonary adenocarcinoma samples are shown in Fig. 6F.

To further investigate the distribution of T cells, we compared CD3⁺ T cell densities in the stromal and tumor regions of two thyroid carcinoma and four lung adenocarcinoma samples. In all cases, the density of CD3⁺ T cells was higher in the stromal region than in the tumor region (Fig. 6G). In thyroid carcinoma, the ratio of stromal to tumor T cell density did not differ markedly between samples with a CXCL12 score of 0 and those with a score of 3 (Fig. 6H). However, in lung adenocarcinoma, samples with higher CXCL12 scores tended to show a higher stromal-to-tumor T cell density ratio.

## Discussion

CAFs are a major component of the TME, and understanding how they suppress T cells function is crucial for cancer immunology^1,23^. In various human solid tumors, limited T cell access to cancer cells has been associated with poor patient prognosis^24^. Recent studies have shown that CAFs attract T cells through the CXCL12/CXCR4 axis. CAF-derived CXCL12 is believed to promote T cell chemotaxis and sequester them within the peritumoral stroma, thereby restricting their infiltration into tumor nests, impairing effective antitumor immunity, and ultimately contributing to poor clinical outcomes^7–9,25^. Although limited, some studies have reported that CAF-derived TGF-β can modulate the CXCL12/CXCR4 axis^13^. In contrast, little is known about canine CAFs, and their interaction with T cell immunity remains largely unexplored. In this study, we evaluated the effect of canine CAFs on T cell migration through the CXCL12/CXCR4 axis and further investigated how CAF-derived TGF-β1 regulates this pathway. Elucidating the factors that control the CXCL12/CXCR4 axis within the TME may provide insights into potential therapeutic strategies for addressing the effect of CAFs on T cell migration.

CXCL12 expression in tumor stroma has been examined in several human cancers, including bladder, gastric and ovarian cancers, and is often associated with poor prognosis^5,7,26,27^. We detected CXCL12 expression in the stroma of eight of the nine canine tumor histo-types examined, with no detectable expression in anal sac gland carcinoma. The association between CXCL12 expression in the tumor stroma and prognosis was not investigated in this study owing to the limited sample size and variable tumor types. Future studies should increase the sample size and focus on specific tumor types to better elucidate the prognostic significance of stromal CXCL12 expression in canine cancers.

CAFs isolated from human tumor tissues secreted higher levels of CXCL12 compared to NFs^5,28^. We found that canine CAFs secreted higher levels of CXCL12 than NFs and that the amount of CXCL12 secreted by CAFs significantly correlated with serum CXCL12 concentrations in the same dogs. While most investigations of CXCL12 have focused on its expression in tissues or cultured cells, some clinical studies have evaluated serum CXCL12 as a potential prognostic biomarker in human medicine. For example, elevated serum CXCL12 levels have been reported in patients with advanced gastric carcinoma and head and neck cancers compared with healthy individuals^29,30^. Although CXCL12 levels can also be elevated in conditions such as inflammation, neural injury, and ischemic diseases^31–33^, the observed correlation in our study shows that increased serum CXCL12 may reflect secretion from CAFs within the TME into the systemic circulation.

This study also identified a regulatory mechanism whereby inhibition of TGF-βRI led to a decrease in CXCL12 secretion by CAFs. This is further supported by the strong positive correlation between CXCL12 and TGF-β1 secretion by CAFs. A previous study of human mammary carcinoma-derived CAFs demonstrated that autocrine TGF-β signaling can upregulate CXCL12 expression through Smad2/3 activation^13^. Consistent with this, our findings indicate that TGF-β signaling may contribute to the regulation of CXCL12 secretion in canine CAFs. Furthermore, given that serum TGF-β levels are higher in tumor-bearing dogs compared with healthy controls^21,34^, and that TGF-β is also secreted by regulatory T cells and cancer cells^35^, TGF-β from various sources within the TME may contribute to the increased CXCL12 secretion by CAFs.

Upregulation of CXCR4 on T cells is associated with various factors, such as hypoxia, TGF-β, vascular endothelial growth factor (VEGF), interleukin (IL)-2, IL-4, IL-7, and IL-15^36,37^. In this study, TGF-β1-derived canine CAFs upregulated CXCR4 on the surface membrane of T cells. A previous human medicine report showed that culture supernatants from CAFs increased CXCR4 expression on T cells and that treatment with an anti-TGF-β neutralizing antibody did not prevent this increase in CXCR4 expression^16^. In the present study, the role of TGF-β1 could have been more directly assessed by using an inhibitor of TGF-β type I receptor ALK5 (also known as activin receptor-like kinase 5), a key component of the canonical TGF-β1 pathway. The results of the in vitro experiments reveal that TGF-β1 secretion by CAFs contributes to the increased CXCR4 expression in cancer-bearing dogs. However, additional factors such as hypoxia and other cytokines may be involved in in vivo conditions. Further studies examining the relationship between serum TGF-β1 concentration, CXCR4 expression in peripheral circulation T cells of cancer-bearing dogs will provide valuable insights. In addition, recent studies have shown that CXCR4 plays a functional role in promoting T cell exhaustion^38^. This shows that upregulation of CXCR4 in the TME may not only mediate chemotaxis but also contribute to impaired antitumor immunity through induction of T cell dysfunction.

The CXCL12/CXCR4 axis has been implicated in the CAF-mediated inhibition of T cell migration. In a human study using CXCL12 knockdown, activated pancreatic stellate cells, which are considered to have a similar function to CAFs, were suggested to promote T cell migration toward stroma and their subsequent retention through CXCL12^9^. Our study demonstrated that the CXCL12/CXCR4 axis facilitates T cell migration induced by canine CAFs, as shown by the inhibitory effects of the CXCR4 antagonist AMD3100. By using AMD3100, it was possible to directly demonstrate that migration is promoted by the effect of CAF-derived CXCL12 on CXCR4 in T cells.

In the co-culture system of T cells, CAFs, and cancer cells, a greater number of T cells adhered to CAFs when co-cultured with RCM-SO, and this adhesion was reduced by CXCR4 inhibition. Co-culture of breast cancer cell lines (MCF-7 and MDA-MB231) and human CAFs resulted in mutual activation, wherein CAFs exhibited an mRNA upregulation of tumor-promoting factors, including CXCL12^39,40^. In this study, a greater number of T cells adhered to CAFs when co-cultured with RCM-SO than with NMTCC. This may be because of differences in the activation of CAFs by cancer cells depending on the cell type and origin.

Although only a limited number of cases were analyzed, we observed accumulation of CD3⁺ T cells in the stromal regions of canine epithelial tumors. This pattern is consistent with findings in human bladder cancer, where CXCL12-positive stroma is associated with increased T cell infiltration^7^. In canine lung adenocarcinoma and thyroid carcinoma, stromal T cell density was higher than in tumor regions, especially in cases with high CXCL12 expression scores. Serial section analysis showed co-localization of CXCL12 expression and CD3⁺ T cells in some cases, but not in all. CD3⁺ T cell accumulation was also observed in CXCL12-negative areas, possibly owing to CXCL12 being a secreted chemokine that diffuses beyond the site of production, making protein detection using IHC challenging. Complementary methods such as in situ hybridization may help clarify local CXCL12 expression. In addition, other chemokines (such as CXCL10, CXCL11, and CCL8) may contribute to T cell localization^16^. Further studies across tumor types and evaluation of CXCR4 expression in stromal T cells are required.

Our findings highlight the critical role of CAFs in regulating T cell migration through the CXCL12/CXCR4 axis, which is modulated by TGF-β1. A similar mechanism has been identified in wound healing, wherein TGF-β enhances CXCL12 secretion from activated fibroblasts and upregulates CXCR4 on macrophages and T cells. This mechanism may promote immune cell recruitment to the wound site^41–43^. In the tumor context, our results show the possibility that CAF-derived CXCL12 contributes to T cell accumulation in the tumor stroma. However, direct evidence that this chemokine inhibits T cell migration into tumor nests remains limited. For example, CD3⁺ T cells were also observed in some CXCL12-negative stroma, and it was technically challenging to directly evaluate the CXCL12-mediated effects of CAFs and tumor cells in vitro. Therefore, further studies are required to clarify the extent to which CXCL12 mediates T cell retention in the TME. This similarity shows that the innate immunomodulatory functions of fibroblasts, which are essential for wound healing, may negatively affect the TME by trapping T cells in the stroma and inhibiting their migration to the tumor cells. Thus, therapeutic strategies targeting the CXCL12/CXCR4 axis or TGF-β1 signaling require careful design, as they may lead to delayed wound healing as a potential systemic side effect.

Cancer immunotherapy, such as immune checkpoint inhibitor or chimeric antigen receptor (CAR) -T cell therapy, has emerged as a promising treatment strategy in recent decades. However, the suppression of T cell immunity through the CXCL12/CXCR4 axis may negatively affect the effectiveness of CAR-T therapy in solid tumors^44^. Previous human studies have reported that CXCR4 is significantly upregulated in patients showing resistance to immunotherapy^45^. Moreover, blockade of CXCR4 has been shown to enhance the efficacy of anti-PD-1/PD-L1 antibodies, leading to increased T cell infiltration within the TME^8^. The findings of the present study indicate that CAFs are involved in T cell recruitment through the CXCL12/CXCR4 axis, which may influence the efficacy of tumor immunotherapy. Elucidating the precise role of this axis could lead to targeted interventions that improve the outcomes of cancer immunotherapy by overcoming CAF-mediated T cell suppression.

Despite the findings, this study had some limitations. One limitation was the difficulty in integrating the original tumor types of CAFs. Given the diverse biological behavior of canine epithelial malignancies, the properties of CAFs may vary between tumor types. In addition, performing a subset analysis of CAFs in this study was difficult. In human medicine, CAFs expressing CXCL12 are known as inflammatory CAFs (iCAFs), which are defined by assessing the expression of multiple markers^46–48^. Human studies have begun to subclassify CAFs based on distinct markers using single-cell analysis^49,50^. Future research should aim to identify markers applicable to canine CAFs and evaluate their functional differences.

**Fig. 7:**
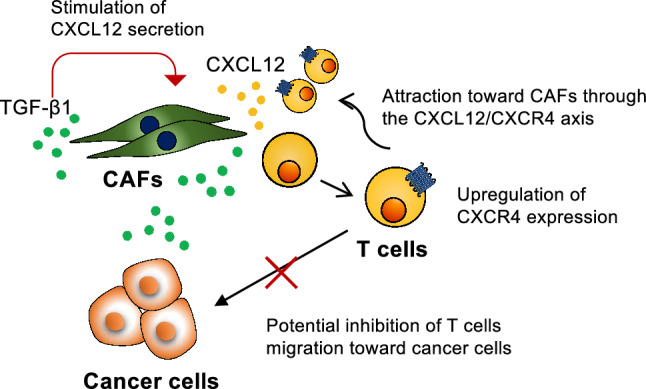
Scheme of CAF-mediated regulation of T cell dynamics through the CXCL12/CXCR4 axis and TGF-β1 signaling. TGF-β1 secreted by CAFs may contribute to the upregulation of CXCR4 on T cells and enhance CXCL12 secretion from CAFs. T cells with increased CXCR4 expression could be attracted to CAF-derived CXCL12 within the TME. This mechanism may potentially interfere with T cell migration toward cancer cells and contribute to immune exclusion.

In conclusion, this study demonstrates that CAFs regulate T cell migration through the CXCL12/CXCR4 axis under the influence of TGF-β1 signaling (Fig. 7). These results underscore the pivotal role of CAFs in the TME, providing valuable insights applicable to both veterinary and human oncology. Our results contribute to the theoretical foundation for novel therapeutic strategies that may involve targeting the CXCL12/CXCR4 pathway modulated by CAFs, with the ultimate goal of improving cancer outcomes across species.

## Methods

### Canine samples

All procedures involving animals were approved by the Animal Experiment Committees of Azabu University (approval no. 220316-26) and conducted in accordance with institutional and AVMA guidelines.

Skin and malignant epithelial tumor tissues were obtained from dogs that underwent surgical resection for epithelial malignancies at the Azabu University Veterinary Teaching Hospital (Kanagawa, Japan). Dogs were premedicated with fentanyl (Termo Corporation, Tokyo, Japan; 5 µg/kg IV), induced with propofol (Viatris Inc., Canonsburg, PA, USA; 6–8 mg/kg IV), and maintained under isoflurane (Zoetis Japan, Tokyo, Japan; 1.5–2.5% in O₂) anesthesia. Post-operative analgesia consisted of fentanyl (2–3 µg/kg/h CRI), ketamine (Fujita Pharmaceutical Co. Ltd., Tokyo, Japan; 0.3 mg/kg/h CRI) and robenacoxib (Elanco Japan, Tokyo, Japan; 2 mg/kg SC), as appropriate. Resected tissues were submitted for histopathological diagnosis, and formalin-fixed specimens were archived at commercial pathology laboratories (Patho Lab Co., Ltd., Shizuoka, Japan; North Lab, Hokkaido, Japan). Peripheral blood was obtained from healthy Beagles (1–6 years old) housed in the Experimental Animal Facility of Azabu University and from client-owned dogs with epithelial malignant tumors treated at the teaching hospital. Blood samples were collected via jugular venipuncture under light sedation with butorphanol (Meiji Seika Pharma Co., Ltd., Tokyo Japan; 0.3 µg/kg SC). No dogs were euthanized solely for research purposes; animals requiring euthanasia for clinical reasons were first rendered deeply anesthetized with propofol (10 mg/kg IV) and then humanely euthanized by intravenous injection of potassium chloride (Maruishi Pharmaceutical Co., Ltd., Osaka, Japan; 1 mEq/kg IV) in accordance with AVMA guidelines and with the owner’s written consent.

Written informed consent was obtained from all owners before inclusion of their animals in the study.

### IHC

To detect the CXCL12 expression, a mouse anti-human/mouse CXCL12 antibody (clone 79018; R&D Systems, Minneapolis, MN, USA)^51^. Notably, canine CXCL12 shares approximately 95% amino acid identity with the human homolog^52^.

Formalin-fixed, paraffin-embedded (FFPE) tissue samples were used for IHC analysis. Freshly cut tissue Sects. (3 μm) were mounted on glass slides, deparaffinized, and subjected to antigen retrieval (CXCL12 and CD3, microwaving at 98 °C for 15 min; α-SMA, autoclaving at 121 °C for 20 min). Primary antibody staining was performed using mouse anti-CXCL12 (diluted 1:100), mouse anti-α-SMA (clone 1A4; diluted 1:1000, Sigma-Aldrich, St. Louis, MO, USA)^53^ and rat anti CD3 (clone CD3-12; diluted 1:300, Bio-Rad Laboratories, Hercules, CA, USA)^54^ monoclonal antibodies, with incubation at 4 °C overnight. Histofine simple stain kit MAX-PO (MULTI) (Nichirei Bioscience Inc., Tokyo, Japan) or Histostar (Rat) (MBL Co., Ltd, Tokyo, Japan) was used as the secondary antibody, and hematoxylin was used as a counterstain. Canine tonsil tissue sections were used as a positive control for CXCL12, vascular smooth muscle was used as an internal positive control for α-SMA, and lymph node was used as a positive control for CD3. Sections without primary antibody staining were used as negative controls. All tissue sections were also stained with standard hematoxylin and eosin for histological examination.

CXCL12 staining intensity in stromal cells was graded as negative, weak, moderate, or strong based on previous human studies that evaluated CXCL12 expression using IHC^5,6^, and the percentage of stained cells was determined by evaluating at least ten high-power fields (200 × magnification) for each tissue section. Tissue specimens were considered positive for CXCL12 when > 10% of stromal cells were stained and negative when ≤ 10% of stromal cells were stained. The evaluation was performed under the guidance of a pathologist. A CXCL12 staining score was assigned to the tumor stroma based on both staining intensity and percentage of positive cells, as follows: 0, no or ≤ 10% of stromal cells stained; 1, > 10% of stromal cells stained with weak intensity; 2, > 10% of stromal cells stained with moderate intensity; and 3, > 10% of stromal cells stained with strong intensity.

Co-localization of CXCL12 expression and CD3^+^ cells in the tumor stroma were evaluated using serial tissue sections. Quantification of CD3^+^ cells in the stromal area was performed using Image J software (U.S. National Institutes of Health, Bethesda, MD, USA). CD3^+^ cell number were counted in ten randomly selected high-power fields withing both the stromal and tumor regions, and the cell density (cell/mm^2^) for each region was calculated.

### Cell culture

NFs and CAFs were isolated from skin and surgically resected tumor tissues using cell dispersion methods^19,55^. Briefly, the tissues were minced into small pieces and digested with 1 mg/mL collagenase IV (Sigma-Aldrich) with 5% bovine serum albumin (BSA; Wako, Osaka, Japan) at 37 °C for 2 h with rotation. Undigested tissues were removed through filtration, and the cells were cultured in Dulbecco’s Modified Eagle’s Medium (DMEM; Wako) supplemented with 10% heat-inactivated fetal bovine serum (FBS; Sigma-Aldrich), 100 units/mL penicillin, and 0.1 mg/mL streptomycin (Wako) at 37 °C with 5% CO_2_. Fibroblasts and tumor cells were separated based on the differential detachment time using 1 mM EDTA-4Na and 0.25% trypsin (Wako). The isolated fibroblasts were cultured and used for the experiments after 1–2 passages following the primary culture^17,55^. NFs and CAFs were cultured in dishes until 80% confluence, after which the media was replaced with RPMI media containing RPMI-1640 with 10% heat-inactivated FBS, 25 mM HEPES (Thermo Fisher Scientific, Waltham, MA, USA), 55 μM 2-mercaptoethanol (Wako), and 100 units/mL penicillin and 0.1 mg/mL streptomycin (Wako). After 24 h of incubation, the culture supernatants were collected as NF-CM and CAF-CM.

cPBMCs were isolated using density gradient centrifugation from peripheral blood samples obtained from healthy beagle dogs and client-owned dogs with epithelial malignant tumors that visited the Azabu University Veterinary Teaching Hospital. The cPBMCs were cultured in RPMI medium at 37 °C with 5% CO_2_.

### Immunocytochemistry (ICC)

For immunofluorescence (IF) analysis of fibroblasts markers, fibroblasts were seeded on chamber slides (eight-well NuncTM Lab-TekTM II Chamber SlideTM System; Thermo Fisher Scientific) at a density of 5.0 × 10^4^ cells/well and cultured for 48 h. The cells were fixed with 4% paraformaldehyde at room temperature (RT) for 1 h, after which they were permeabilized with 0.5% Triton X-100 (Wako) in phosphate-buffered saline (PBS, Wako) for 15 min and blocked with 1% BSA for 1 h. Immunostaining was then performed using a rabbit anti-vimentin antibody (Clone SP20; 1:1000, Abcam, Cambridge, UK) as a marker for mesenchymal cells, a mouse anti-cytokeratin antibody (clone AE1/AE3; 1:200, Novus Biologicals, Centennial, CO, USA) as a marker of epithelial cells, a mouse anti-α-SMA (clone 1A4; 1:500, Thermo Fisher Scientific), and a mouse anti-CXCL12 antibody (clone 79,018; 1:40, R&D Systems), all diluted in PBS and incubated at 4 °C overnight. The cells were subsequently incubated with a goat anti-rabbit (Alexa-Fluor-594, Cell signaling technology, Danvers, MA, USA) or goat anti-mouse (Alexa-Fluor-488, Thermo Fisher Scientific) secondary antibody at RT for 1 h, followed by nuclei counterstaining with DAPI (Dojindo Laboratories, Kumamoto, Japan) at RT for 15 min. Coverslips were mounted on the glass slides, and images were obtained using a confocal microscope (TCS SP5Ⅱ; Leica, Wetzlar, Germany).

To verify that vascular endothelial cells were not present, the negative expression of CD31, a marker for endothelial cells, was assessed. CAFs and NFs were detached using trypsin and embedded in iPGell (Genostaff, Tokyo, Japan) according to the manufacturer’s instructions. The gel-embedded samples were then paraffin-embedded and processed for IHC using the same protocol used for tissue sections. Briefly, FFPE blocks were sectioned at 3 μm, mounted on glass slides, deparaffinized, and subjected to antigen retrieval by autoclaving at 121 °C for 20 min. A ready to-use mouse monoclonal anti-CD31 (Clone JC70A; Dako, Glostrup, Denmark)^53^ was applied, followed by detection using the Histofine Simple Stain MAX-PO (MULTI) kit. Canine hemangiosarcoma tissue was used as a positive control.

### Cytokine quantification

The concentration of CXCL12 in cell culture supernatants and serum was measured using a Canine Stromal Cell-Derived Factor 1 ELISA kit (MBS2606294; MyBioSource, San Diego, CA, USA). To examine the correlation between CXCL12 concentrations in culture supernatants and serum, we used serum samples collected from the same dogs prior to surgical resection of the tumors from which the CAFs were derived. The concentration of TGF-β1 in cell culture supernatants was measured using a Human/Mouse/Rat/Porcine/Canine TGF-β1 Quantikine ELISA kit (DB100C; R&D Systems). All assays were performed according to the manufacturer’s instructions. Absorbance was measured at 450 nm using an iMark™ Microplate Absorbance Reader (Bio-Rad Laboratories).

### Flowcytometry

To detect the CXCR4 expression on the T cell membrane, a biotin-conjugated mouse anti-human CXCR4 antibody (clone 12G5; Thermo Fisher Scientific) was used. To evaluate antibody specificity and cross-reactivity in canine cells, we performed serial dilution flow cytometry using cPBMCs, which showed a concentration-dependent decrease in CXCR4 mean fluorescence intensity (MFI) (Supplementary Fig. S5A). In addition, a CXCL12 competition assay demonstrated a ligand dose-dependent reduction in CXCR4 staining (Supplementary Fig. S5B), supporting the specific binding of clone 12G5 to canine CXCR4. Notably, canine CXCR4 shares approximately 95% amino acid identity with the human homolog^52^.

cPBMCs were washed twice in fluorescence-activated cell sorting (FACS) buffer (1% FBS and 0.1% NaN₃ in PBS). T cells were first stained with the biotin-conjugated mouse anti-human CXCR4 antibody for 30 min at 4 °C. A negative control was stained with an isotype control antibody (clone eBM2a; Thermo Fisher Scientific). After washing twice, cells were stained with a Pe-Cy7 conjugated streptavidin (Thermo Fisher Scientific) for 30 min at 4 °C to detect the biotin-labeled antibodies. In addition, cells were stained with the following directly fluorochrome-conjugated anti-canine antibodies: FITC-conjugated mouse anti-dog CD3 (clone CA17.2A12; Bio-Rad Laboratories), PE-conjugated anti-CD4 antibody (clone YKIX302.9, Bio-Rad Laboratories) and APC-conjugated rat anti-dog CD8 (clone YCATE 55.9; Thermo Fisher Scientific). The PE-conjugated anti-dog CD4 antibody was not included in the analysis for some of the clinical samples. To exclude dead cells, 7AAD (BioLegend, San Diego, CA, USA) was used. Data were acquired using a BD FACSCelesta cell analyzer (BD Biosciences, San Jose, CA, USA) and analyzed using FlowJo software (version 10; Treestar, Ashland, OR, USA). Flow cytometry gating schemes are shown in Supplementary Fig. S6.

### TGF-β1 treatment assay for evaluating the effects on the CXCL12/CXCR4 axis

To evaluate the effect of TGF-β1 on the CXCL12/CXCR4 axis, CAFs were cultured with or without 5 μM of SB431542 (Cayman Chemical, Ann Arbor, MI, USA), a selective inhibitor of TGF-β type I receptor ALK5 (TGF-βRI inhibitor), effectively inhibits the TGF-β pathway^56^. After 72 h of incubation, the media was replaced with 2% FBS DMEM. The concentration of CXCL12 in the 24 h incubation culture supernatant was measured using ELISA. The experiment was conducted using only the samples that yielded sufficient viable cells for analysis.

cPBMCs obtained from healthy dogs were stimulated using Dynabeads M-280 Tosylactivated (DB14204; Thermo Fisher Scientific) coated with anti-dog CD3 and anti-dog CD28 antibodies (clone 1C6; Absolute Antibody, Upper Heyford, UK) and cultured with 5 ng/mL recombinant human TGF-β1 (PeproTech, Cranbury, NJ, USA) for 5 days. Recombinant human TGF-β1 was used because the amino acid sequence of canine TGF-β1 is 100% identical to that of human TGF-β1. Experiments were performed with three different donors, and the data shown represent the average across these three biological replicates. CXCR4 expression was then evaluated through flow cytometry. Similarly, the stimulated cPBMCs were cultured with NF-CM or CAF-CM with or without 5 μM of SB431542 for 5 days, and CXCR4 expression was evaluated.

### Migration assay

A Boyden chamber assay was performed to evaluate the effect of the CXCL12/CXCR4 axis on the migration of canine CD8^+^ T cells. Canine CD8^+^ T cells were magnetically isolated from cPBMCs using an EasySep™ Positive Selection Kit Ⅱ (STEMCELL, Vancouver, Canada) and cultured overnight to promote CXCR4 expression (Supplementary Fig. S7). The separation efficiency and CXCR4 expression were confirmed to be > 90% prior to use. Canine CD8^+^ T cells, either untreated or pretreated for 1 h with 4 µM AMD3100 (Abcam), a CXCR4 antagonist, were added to the upper compartment of the cell culture inserts (pore size, 8 μm; Corning Inc, Corning, NY, USA) (Fig. 6A). The lower compartment was supplemented with either 100 ng/ml of recombinant human CXCL12 (R&D Systems) or CAFs-CM. After 1.5 h of incubation, the number of migrated cells in the lower compartment was counted using a hemocytometer.

### Co-culture assay

To evaluate the interaction between T cells, canine CAFs, and cancer cell lines, co-culture experiments were performed. CAFs and canine epithelial tumor cell lines (mammary gland tumor, RCM-SO; transitional cell carcinoma, NMTCC) were seeded separately in 6-cm petri dishes using a cell culture insert (flexiPERM disc; Sarstedt, Osaka, Japan) to maintain spatial separation and cultured for 24 h (Fig. 6D). The insert was removed prior to the co-culture.

Canine CD5^+^ T cells were magnetically isolated from cPBMCs using an EasySep™ Positive Selection Kit Ⅱ and cultured overnight to enhance CXCR4 expression, as described above. The separation efficiency and CXCR4 expression were confirmed to be > 90% prior to use. The CD5^+^ T cells were then seeded onto CAFs and tumor cells plated dishes at a density of 1.0 × 10^6^ cells/dish in the presence or absence of 4 μM of AMD3100. After 24 h incubation, non-adherent T cells were removed by washing the dishes three times with PBS. Five randomly selected fields in CAFs or tumor cell areas were imaged under a phase-contrast microscope (BZ-X; KEYENCE, Osaka, Japan).

T cell adhesion was quantified using Hybrid Cell Count Software (BZ-H3C; KEYENCE). First, the total area covered by CAFs or tumor cells was measured. Next, small, round cells located on top of the adherent cell layer were identified and counted based on size and circularity features. The density of adherent T cells was expressed as the number of T cells per cm^2^ of adherent cell area. Two independent researchers performed image analysis in a blinded fashion.

### Statistical analysis

Data are presented as the mean ± standard deviation (SD) or the median with interquartile range (IQR). Statistical analyses were performed using GraphPad Prism 10 (GraphPad Software Inc., Boston, MA, USA). Normality was assessed using the Shapiro–Wilk test with the significance level set at 0.05. For comparisons between two groups, Welch’s t-test was used for normally distributed data and Mann–Whitney U test was used for non-normally distributed data. For comparisons among at least three groups, one- or two-way analysis of variance (ANOVA) was performed, followed by an appropriate post hoc test: Tukey’s test for normally distributed data and Dunn’s test for non-normally distributed data. Correlations between two variables were assessed using Spearman’s rank correlation coefficient. Statistical significance was set at *P* < 0.05.

## Supplementary Information

Supplementary Information.

## Data Availability

All data generated or analyzed during this study are included in this published article and its supplementary information files. Further inquiries can be directed to the corresponding author.

## Declarations

### ARRIVE guidelines

The study is reported in accordance with the ARRIVE guidelines.
