# Supplementary material for: Exploring the effect of canine cancer-associated fibroblasts on T cell dynamics through the CXCL12/CXCR4 axis modulated by TGF-β1
Source: Sci Rep. 2025 Aug 23;15:31050. doi: 10.1038/s41598-025-16312-x (PMC12375069; doi:10.1038/s41598-025-16312-x)
Supplement: Supplementary file 1 — Supplementary Information. [file 41598_2025_16312_MOESM1_ESM.pdf]

Supplementary Fig. S1

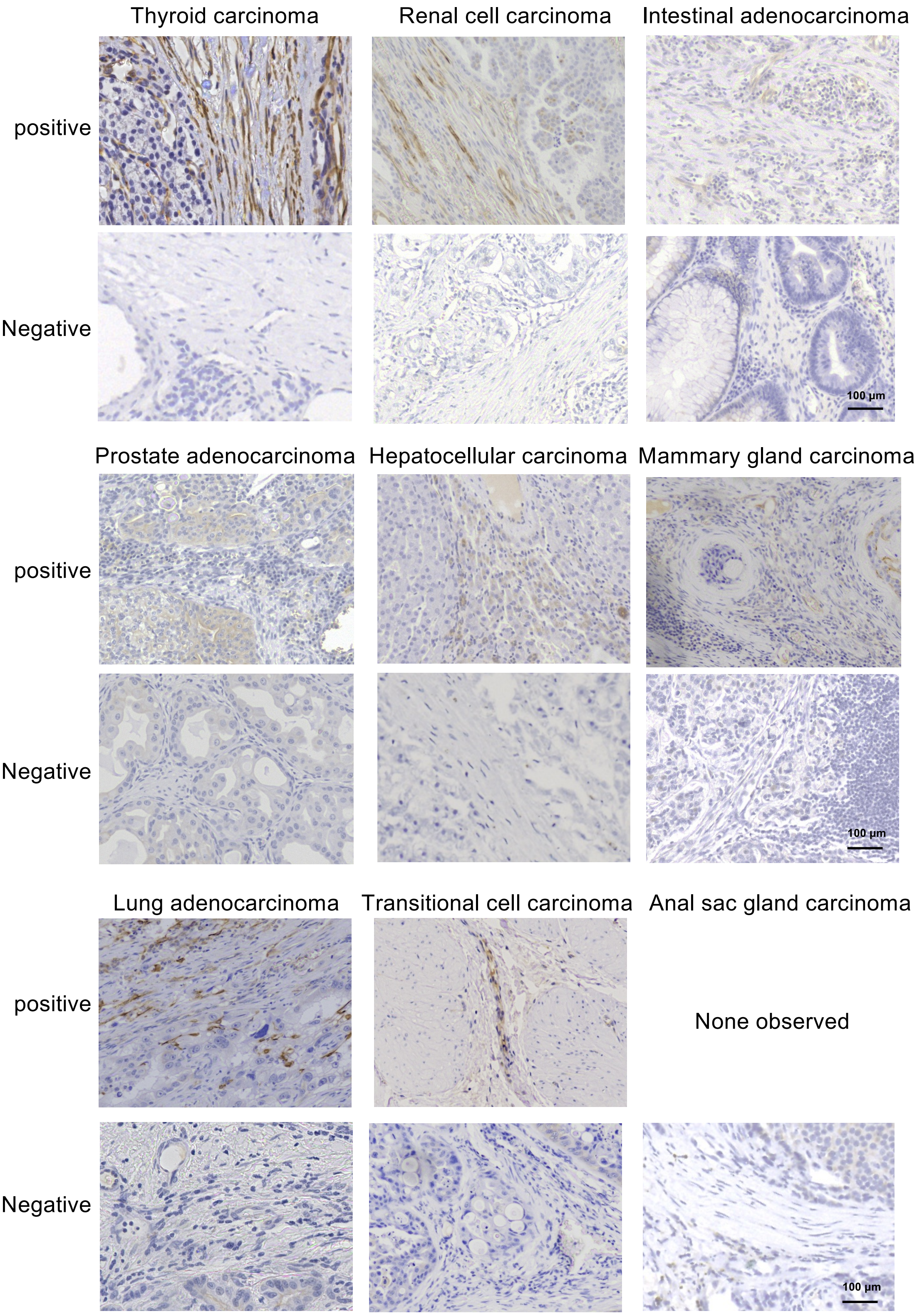

Representative C-X-C motif chemokine ligand 12 (CXCL12) immunohistochemical staining in various canine epithelial malignant tumors. For each of the 9 tumor histo-types, thyroid carcinoma, renal cell carcinoma, intestinal adenocarcinoma, prostate adenocarcinoma, hepatocellular carcinoma, mammary gland carcinoma, lung adenocarcinoma, transitional cell carcinoma, and anal sac gland carcinoma, representative CXCL12-positive and -negative stromal expression patterns are shown. Staining was performed on formalin-fixed, paraffin-embedded tumor sections. Scale bars = 100 µm.

Supplementary Fig. S2

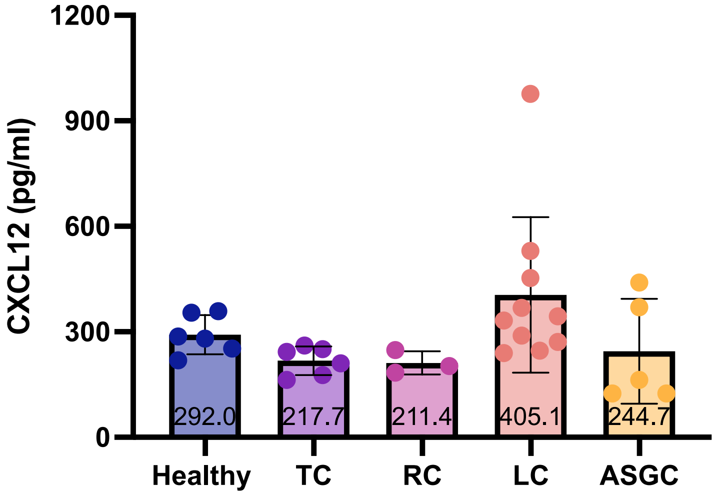

CXCL12 concentration in the serum of healthy and cancer-bearing dogs. Serum concentrations of CXCL12 were quantified using enzyme-linked immunosorbent assay (ELISA). Data are presented as mean ± standard deviation (SD). Healthy dogs (*n* = 6); thyroid carcinoma (TC; *n* = 6); renal cell carcinoma (RC; *n* = 3); lung adenocarcinoma (LC; *n* = 10); anal sac gland carcinoma (ASGC; *n* = 5).

Supplementary Fig. S3

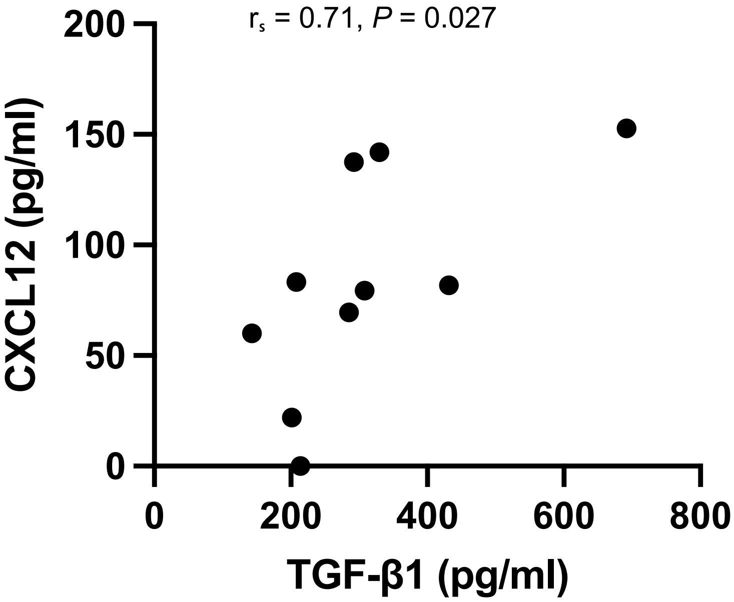

Correlation between CXCL12 and transforming growth factor beta 1 (TGF-β1) concentration secreted from cancer-associated fibroblasts (CAFs)

Correlation between CXCL12 and TGF-β1 concentrations in cancer-associated fibroblast culture medium is shown in the scatter plot with the Spearman's rank correlation coefficient (r_s) and *P* value.

Supplementary Fig. S4

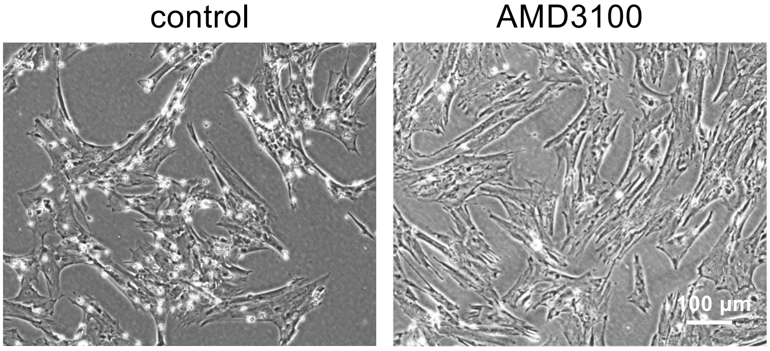

Microscopic image of T cells attached to CAFs)

C-X-C chemokine receptor 4 (CXCR4)-positive T cells were co-cultured with CAFs and tumor cells for 24 h. Representative images show small, spherical T cells adhered to CAFs. The number of T cells attached to CAFs was reduced in the presence of AMD3100, a CXCR4 antagonist.

Supplementary Fig. S5

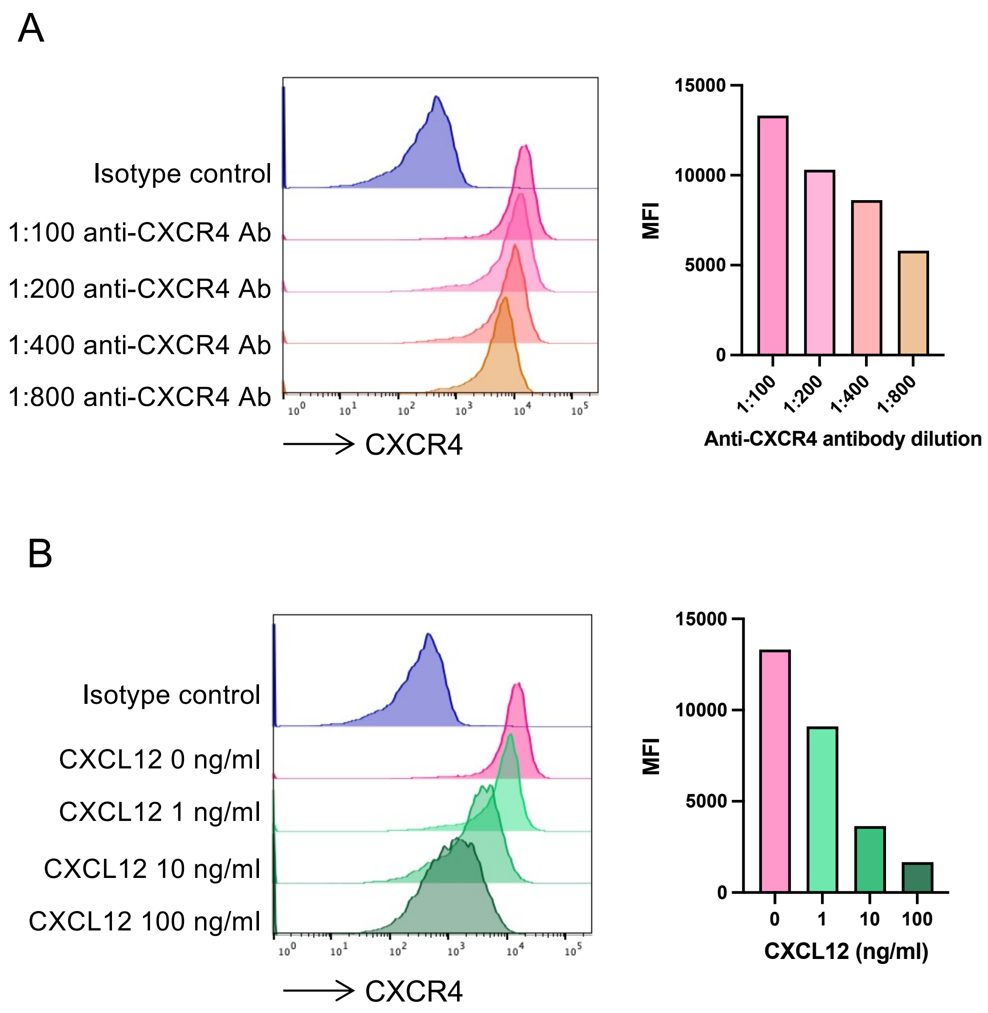

Validation of the cross-species reactivity and binding specificity of the anti-CXCR4 antibody to canine T cells.

(A). Flow cytometric analysis of CXCR4 expression in canine CD3^{+} T cells stained with anti-CXCR4 antibody at serial dilutions (1:100 to 1:800). Mean fluorescence intensity (MFI) decreased with antibody dilution, indicating concentration-dependent antigen recognition.

(B). CXCL12 competition assay. Canine T cells were co-cultured with increasing concentrations of CXCL12 (0, 1, 10, and 100 ng/mL), the ligand of CXCR4, for 2 h prior to staining. CXCR4 MFI was reduced in a ligand dose-dependent manner, suggesting specific binding of the antibody to the CXCR4 receptor.

Supplementary Fig. S6

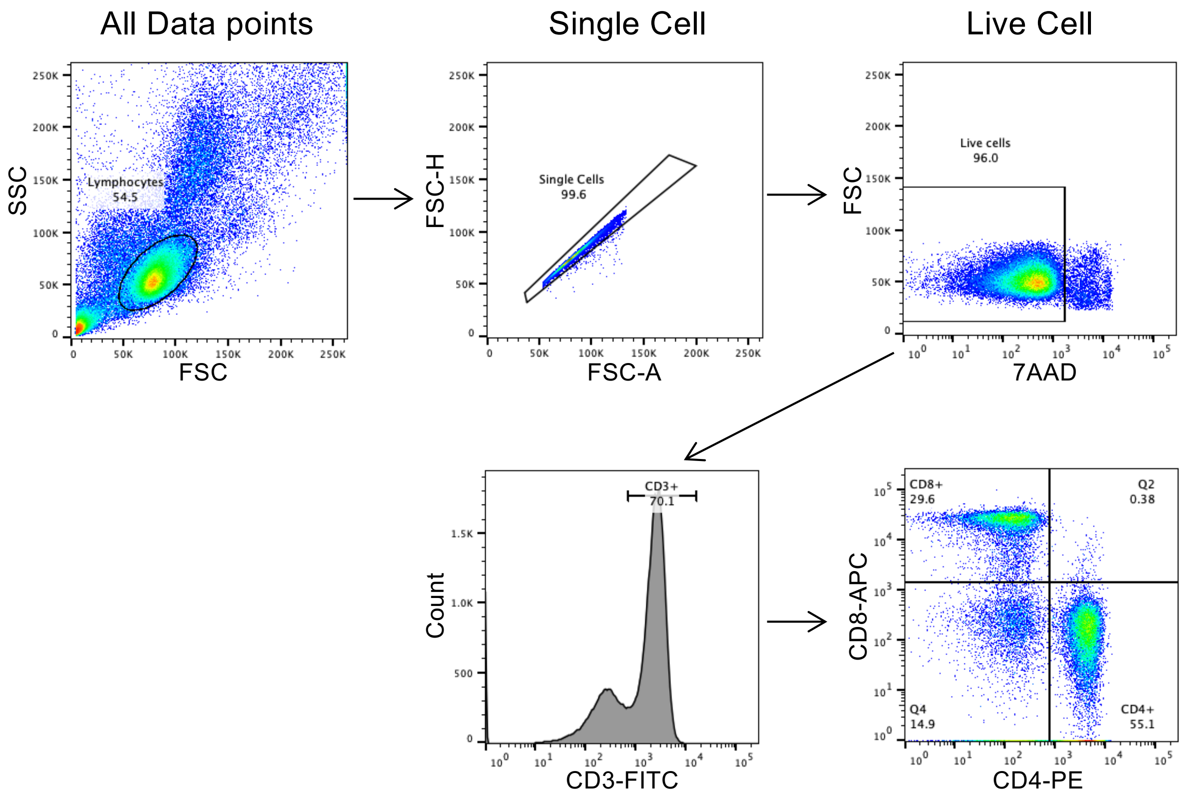

Gating strategy for identification of canine T cell subsets by flow cytometry. Lymphocytes were first gated based on forward scatter (FSC) vs. side scatter (SSC), followed by exclusion of doublets using FSC-A (area) vs. FSC-H (height), and dead cells by 7AAD staining. $CD3^{+}$ T cells were then identified and further characterized into $CD4^{+}$ and $CD8^{+}$ subsets. Numbers indicate the percentage of cells within the parent gate.

Supplementary Fig. S7

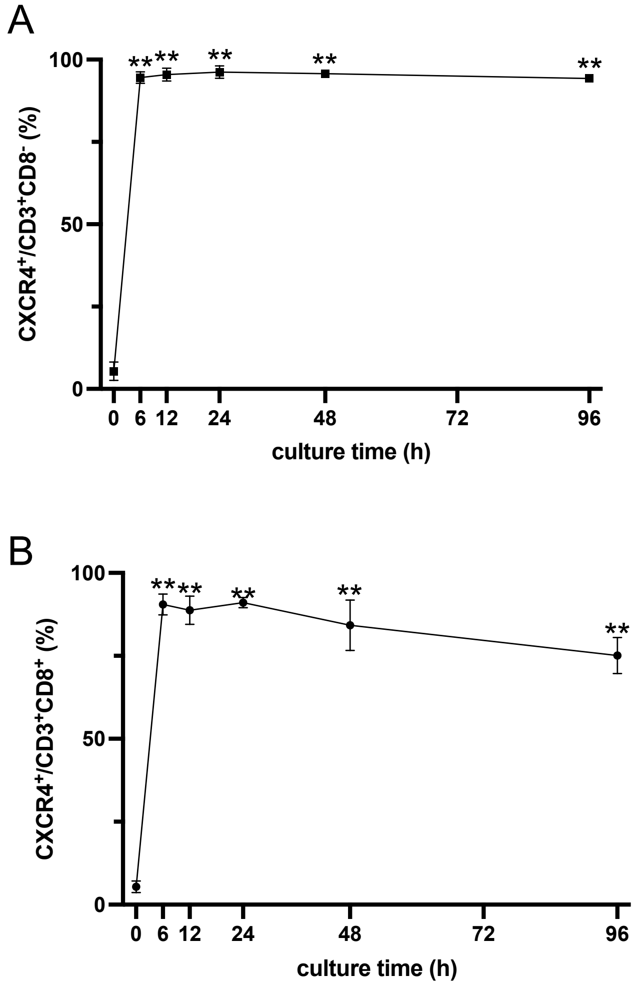

Increase in CXCR4 expression on canine T cells after incubation

(**A**) CXCR4 expression on canine $CD3^+CD8^-$ T cells over time. (**B**) CXCR4 expression on canine $CD3^+CD8^+$ T cells over time. Before culture, CXCR4 expression on T cells isolated from healthy dogs was less than 10%, which rapidly increased after incubation at 37 °C with 5% CO_2. High CXCR4 expression levels were maintained for up to 96 h. Data are presented as the mean ± SD (n = 3). Asterisks indicate a significant difference compared with 0 h. $**P < 0.01$.

Supplementary Table S1. Summary of canine epithelial tumor samples

No.	Breed	Age	Sex[※a]	Diagnosis[※b]
Dog 1	Italian Greyhound	9y	MC	RC
Dog 2	Bichon Frize	11y	MC	PC
Dog 3	Australian Labradoodle	7y	MC	TC
Dog 4	Miniature Dashshund	9y	FS	TC
Dog 5	Maltese	12y	FS	LC
Dog 6	Chihuahua	14y	FS	LC
Dog 7	Standard Poodle	11y	CM	LC
Dog 8	Pekingese	9y	CM	LC
Dog 9	Mix Breed	15y	M	ASGC
Dog 10	Miniature Dashshund	14y	M	ASGC

※a MC: male castrated, M: male, FS: female spayed
※b RC: renal cell carcinoma, PC: prostate adenocarcinoma, TC: thyroid carcinoma, LC: lung adenocarcinoma, ASGC: anal sac gland carcinoma.
